# Unmet healthcare needs among adults with disabilities in South Korea: a nationally representative analysis using the behavioral model

**DOI:** 10.1186/s12889-026-28057-x

**Published:** 2026-06-26

**Authors:** Eun-Mi Seol, Soo-Hyun Nam

**Affiliations:** 1 College of Nursing, Inje University, Gimhae, Republic of Korea; 2 School of Nursing Science, Gyeongkuk National University, 1375 Gyeongdong-ro, Andong, 36729 Republic of Korea

**Keywords:** Unmet healthcare needs, Disability, Healthcare access, Health equity, Andersen's behavioral model, South Korea

## Abstract

**Background:**

Unmet healthcare needs are a critical indicator of health inequities. Despite near-universal health insurance coverage in South Korea, people with disabilities experience substantial barriers to healthcare access. This study identified factors associated with unmet healthcare needs among people with disabilities in South Korea.

**Methods:**

This cross-sectional study used data from the 2023 Korea National Survey of People with Disabilities (KNSPD 2023), a nationally representative survey. The survey employed two-stage stratified cluster sampling, yielding an analytic sample of 6,772 adults with registered disabilities (weighted *N* = 2,630,374). Applying Andersen’s Behavioral Model, complex-sample logistic regression examined predisposing, enabling, and vulnerability factors associated with unmet healthcare needs in the past 12 months.

**Results:**

Overall, 17.3% of people with disabilities reported unmet healthcare needs. Transportation difficulties (36.5%) and financial cost (27.8%) accounted for nearly two-thirds of reported barriers. Among enabling factors, lack of health insurance emerged as the strongest predictor of unmet healthcare needs. Physical disability and poor self-rated health were also associated with substantially increased odds of unmet healthcare needs. Individuals with severe disabilities had lower odds of unmet healthcare needs than those with mild disabilities, suggesting potential gaps in support for people with less severe functional limitations. Women with physical disabilities showed particularly elevated odds of unmet healthcare needs.

**Conclusions:**

Despite universal insurance coverage, substantial unmet healthcare needs persist among people with disabilities in South Korea, driven primarily by transportation and financial barriers. Policy priorities should include expanding paratransit services, reducing out-of-pocket costs, targeting insurance enrollment assistance to the uninsured, addressing service gaps for mild disabilities, and implementing sex-specific interventions for women’s compounded barriers. Healthcare access equity requires moving beyond coverage expansion to address structural accessibility barriers.

**Supplementary Information:**

The online version contains supplementary material available at https://doi.org/10.1186/s12889-026-28057-x.

## Introduction

Ensuring equitable access to essential healthcare services is a central objective of public health systems worldwide [1]. In South Korea, the expansion of the National Health Insurance (NHI) system has enabled near-universal health coverage to provide equitable access regardless of socioeconomic status [2]. Despite this comprehensive coverage, people with disabilities continue to face significant challenges in obtaining timely and appropriate care. This is particularly concerning given that their healthcare needs are generally greater than those of the non-disabled population [3, 4]. The persistent gap between near-universal insurance coverage and actual access to needed healthcare services highlights a critical limitation of the current healthcare system and underscores ongoing inequities.

Multiple structural and interpersonal barriers contribute to these disparities. These include insufficient disability-friendly infrastructure, limited provider training on disability-specific needs, and fragmented coordination between healthcare and social welfare systems [5]. Such barriers translate into tangible difficulties—restricted physical accessibility, long waiting times, high out-of-pocket costs, and challenges navigating complex healthcare pathways [6, 7]. International evidence mirrors these patterns: for example, 40% of Europeans with disabilities report unmet medical needs, compared with 17% among those without disabilities [8], and similar disparities have been documented in the United States [9]. In South Korea, approximately 32.4% of individuals with disabilities experience unmet healthcare needs [10, 11], with economic burden, accessibility barriers, and time constraints identified as primary reasons [12]. These findings emphasize the urgency of addressing inequities even within a universal health coverage system.

Unmet healthcare needs are defined as the gap between healthcare services that individuals perceive as necessary and those they actually receive [13]. This construct encompasses three interrelated components: (1) perceived need—individual recognition of a health problem requiring professional care; (2) healthcare-seeking behavior—intention or attempt to access services; and (3) barriers to access—structural, financial, and attitudinal factors preventing service receipt [14, 15].

Within healthcare access frameworks, unmet needs arise from mismatches between population health needs and healthcare system capacity. Penchansky and Thomas conceptualized access as comprising five dimensions: availability, accessibility, affordability, accommodation, and acceptability [16]. Unmet healthcare needs thus reflect not only individual-level circumstances but also broader system-level failures to adequately address one or more of these dimensions.

This issue is particularly consequential for people with disabilities, who typically have higher healthcare needs and face multiple barriers in accessing services not designed with disability accommodation in mind. In South Korea, 84.8% of adults with disabilities have at least one chronic condition, with an average of 2.5 comorbidities [17]. Identifying the factors associated with unmet needs in this population is essential for understanding system failures and informing disability-inclusive policy reforms. While previous research has examined unmet needs in the general Korean population [12], systematic examination among adults with disabilities using nationally representative data and comprehensive theoretical frameworks remains limited.

The Behavioral Model for Vulnerable Populations provides a framework for understanding factors that influence healthcare access and utilization [18]. This model categorizes factors into three domains: (1) predisposing factors—demographic and social characteristics (age, sex, education, marital status) that exist prior to healthcare need; (2) enabling factors—resources and structural conditions that facilitate or hinder access (income, insurance, employment, geographic location, functional limitations); and (3) vulnerability factors—health-related characteristics that heighten need for services (disability type and severity, chronic conditions, functional dependence, health behaviors, mental health). Applying this framework to people with disabilities allows systematic examination of how individual characteristics, resource availability, and health status interact to shape healthcare access experiences. This framework is well-suited for examining unmet healthcare needs, as it captures both individual-level factors and structural barriers to healthcare access.

Using nationally representative data, this study aims to provide a theoretically grounded and policy-relevant analysis of unmet healthcare needs among adults with disabilities in South Korea. Specifically, this study aims to: (1) estimate the prevalence and reasons for unmet healthcare needs; (2) identify predisposing, enabling, and vulnerability-related factors associated with unmet needs; and (3) examine differences by sex.

## Materials and methods

### Data source and study sample

This cross-sectional study used data from the 2023 National Survey of Persons with Disabilities (KNSPD 2023), a nationally representative survey conducted by the Korea Institute for Health and Social Affairs in collaboration with the Ministry of Health and Welfare [17]. This nationwide survey has been administered every five years since 1980 to assess the living conditions and welfare needs of people with disabilities and to support evidence-based policy development [11].

The survey employed a two-stage stratified cluster sampling design. Primary sampling units (PSUs) were administrative regions (eup, myeon, and dong), and individuals aged ≥ 18 years with registered disabilities were randomly selected within each PSU. All variables used in this study, including the outcome and independent variables, were derived from the 2023 National Survey of Persons with Disabilities.

Survey weights were applied in all analyses to account for the complex sampling design and to ensure nationally representative estimates. The weights incorporate three components: (1) base sampling weights (inverse probabilities of selection at both sampling stages), (2) non-response adjustment by age, sex, disability type, and region, and (3) post-stratification to the 2023 national disability registry population totals. These weights allow the analytic sample of 8,000 participants to represent approximately 2.63 million adults with registered disabilities in South Korea.

Of the 8,000 participants, 228 (2.9%) had missing data on the outcome variable and 1,000 (12.5%) had missing data on covariates (primarily income and smoking status), resulting in a final analytic sample of 6,772 (unweighted) or 2,630,374 (weighted).

Participants were included if they were aged ≥ 18 years and registered in the national disability registry. Individuals were excluded if they had missing data on the outcome variable (unmet healthcare needs) or key covariates (e.g., income and smoking status).

Comparison of participants with complete versus incomplete data showed no statistically significant differences in unmet healthcare needs prevalence (17.3% vs. 17.8%, *p* = 0.42), disability severity distribution (*p* = 0.38), or sex distribution (*p* = 0.56), suggesting minimal selection bias. Detailed comparisons are presented in Supplementary Table S1.

Variance estimates were calculated using Taylor series linearization, accounting for stratification and clustering, implemented using the Complex Samples module in IBM SPSS Statistics version 29.0.

### Dependent variable

The primary outcome was unmet healthcare needs, defined as the gap between healthcare services individuals perceive as necessary and those actually received [13]. Unmet healthcare needs were operationally assessed using a single survey item asking whether participants experienced times when they needed medical care but could not receive it in the past 12 months, consistent with international surveillance approaches [13, 14]. Respondents answered “yes” or “no” to the question:

In the past year, were you unable to visit a medical institution when you wanted to?

For respondents who reported unmet healthcare needs, reasons were assessed using a predefined single-choice item asking participants to select the main reason for not receiving needed healthcare. Because respondents selected only one primary reason, the reported reasons and categories are mutually exclusive and sum to 100% among individuals reporting unmet healthcare needs.

The twelve original survey response options were subsequently grouped into three access-related dimensions—accessibility, availability, and acceptability—for descriptive interpretation, informed by Chen and Hou [14] and the healthcare access framework proposed by Penchansky and Thomas [16]. Because these categories were applied post hoc rather than embedded in the original questionnaire design, they should be interpreted as pragmatic descriptive classifications rather than validated theoretical constructs.

- Accessibility (physical and financial access): transportation difficulties, financial cost.
- Availability (service supply): services not available in the area, services unavailable when needed, waiting time too long.
- Acceptability (personal and attitudinal factors): too busy, personal or family caregiving responsibilities, dislike or fear of healthcare providers, did not know where to seek care, perceived inadequacy of care, other.

This single-item self-reported measure was selected because it has been consistently used in Korean national health surveys (Korea National Health and Nutrition Examination Survey and Korea Community Health Survey) since 2008 [12] and in international surveillance systems, including the Canadian Community Health Survey [14], European Health Interview Survey [19], and US National Health Interview Survey [9]. This approach enables standardization across surveys, reflects patient-centered perceptions of access barriers, and minimizes respondent burden for population-level assessment.

While alternative approaches such as multidimensional validated instruments or provider-assessed need offer advantages in capturing specific dimensions, they were not available in this secondary dataset. Approximately 12% of responses were provided by proxies due to cognitive or communication impairments. Sensitivity analysis excluding proxy-reported cases (*n* = 5,956; 88% of sample) yielded substantively identical results, with all odds ratios within ± 5% of the main analysis. Detailed results of the proxy-response sensitivity analysis are presented in Supplementary Table S2. Limitations of this measurement approach are discussed in the Limitations section.

### Independent variables

Independent variables were classified according to the Behavioral Model for Vulnerable Populations, which includes predisposing, enabling, and vulnerable factors.

#### Predisposing factors

Predisposing variables included demographic characteristics not directly related to medical need:

- Age: 19–34, 35–49, 50–64, ≥ 65 years
- Sex: male, female
- Marital status: married and cohabiting; married but not cohabiting, bereaved, or divorced; unmarried
- Education: elementary school or lower, middle school, high school, college or higher

#### Enabling factors

Enabling factors capture individual and contextual resources that facilitate or hinder healthcare access:

- Region: Seoul; metropolitan areas (Busan, Daegu, Incheon, Gwangju, Daejeon, Ulsan, Sejong, Jeju); other provinces
- Employment status: employed, unemployed
- Income: quartiles (1Q–4Q; lowest to highest)
- Medical insurance type: National Health Insurance, Medicaid, no insurance/do not know

Medicaid in South Korea is a government-funded insurance program for low-income individuals who meet eligibility criteria.

#### Vulnerability factors

Vulnerability-related characteristics included disability-specific and health-related variables:

- Going out alone: possible, impossible
- ADL and IADL dependency: Functional limitations were assessed using ADL and IADL measures from the 2023 National Survey of Persons with Disabilities [17]. Both use 4-point scale (1 = completely independent to 4 = completely dependent) and were analyzed as continuous variables in the main analysis. Sensitivity analyses using categorical coding (any dependency vs. complete independence) yielded consistent results; detailed findings are presented in Supplementary Table S3.
- Disability severity: classified as mild (grades 4–6) or severe (grades 1–3) based on the Korean disability classification system.
- Disability type: categorized as physical disability or intellectual disability. Under Korean law, disabilities are classified into 15 statutory categories, which were grouped into these two categories for analysis.
- Smoking status: non-smoker, past-smoker, current-smoker
- Alcohol consumption: never, < 1 time/month, 1–4 times/month, ≥ 5 times/month
- Exercise: yes, no
- Stress level: high, moderate, low, none
- Self-rated health: very good/good, moderate, bad/very bad
- Depression: yes, no

### Statistical analyses

All analyses were conducted using IBM SPSS Statistics version 29.0 (IBM Corp., Armonk, NY, USA) with the Complex Samples module. Analyses used weighted data and accounted for the complex survey design, including stratification, clustering, and sampling weights.

Descriptive statistics were used to summarize participant characteristics. Categorical variables were presented as unweighted frequencies and weighted percentages, while continuous variables were presented as weighted means with standard errors.

Differences between individuals with and without unmet healthcare needs were examined using design-based chi-square tests for categorical variables and design-based t-tests for continuous variables.

To identify factors independently associated with unmet healthcare needs, multivariable complex-sample logistic regression analysis was performed. All predictor variables—predisposing factors (age, sex, marital status, education), enabling factors (region, employment, income, insurance, functional limitations), and vulnerability factors (disability severity, disability type, health behaviors, health status)—were simultaneously included in the regression model. Results are presented as adjusted odds ratios (ORs) with 95% confidence intervals (CIs), representing the association between each predictor and unmet healthcare needs while controlling for all other variables in the model. Statistical significance was defined as *p* < 0.05.

Multicollinearity was assessed using variance inflation factors (VIFs), and all values were below 3.0, indicating minimal multicollinearity; detailed diagnostics are presented in Supplementary Table S4. ADL and IADL dependency scores were modeled as continuous variables in the main analysis. Sensitivity analyses using alternative coding approaches yielded substantively similar results, with detailed findings presented in Supplementary Table S3.

### Ethics statement

This study was conducted in accordance with the principles of the Declaration of Helsinki and relevant ethical guidelines for secondary data analysis. Ethical approval was obtained from the Institutional Review Board of Gyeongkuk National University (IRB No. 2507-172-1659). The dataset used in this study was fully de-identified and contained no personally identifiable information. As the data were derived from de-identified national survey data and analyzed retrospectively, the requirement for informed consent from individual participants was waived by the IRB in accordance with institutional and national regulations. Data were used solely for research purposes in accordance with ethical guidelines.

## Results

### Study population characteristics

A total of 8,000 participants (unweighted sample) were included in the analysis, representing 2,630,374 adults with registered disabilities in South Korea (weighted population). Physical disabilities constituted the largest proportion of the weighted population (44.4%; *n* = 1,169,082), followed by auditory impairment (16.4%), visual impairment (9.5%), brain impairment (9.2%), and intellectual disability (7.9%).

The distribution of disability types differed by severity. Among individuals with mild disabilities, physical impairments were most common (56.6%), followed by auditory (20.3%) and visual (12.0%) impairments. Among those with severe disabilities, intellectual disability (22.5%), physical impairment (21.7%), and brain impairment (14.8%) were most prevalent (Table 1).

**Table 1:** Types and severity of disability (*N* = 2*630*,374)

Type of disability	Disability		
	Mild, *n* (%)	Severe, *n*(%)	Total
Physical impairment	970,290 (56.6)	198,792 (21.7)	1,169,082 (44.4)
Brain impairment	106,681 (6.2)	135,723 (14.8)	242,404 (9.2)
Visual impairment	205,478 (12.0)	44,488 (4.8)	249,966 (9.5)
Auditory impairment	348,455 (20.3)	82,206 (9.0)	430,661 (16.4)
Linguistic impairment	13,893 (0.8)	9,326 (1.0)	23,219 (0.9)
Intellectual disability	0 (0.0)	206,636 (22.5)	206,636 (7.9)
Developmental disability	0 (0.0)	38,119 (4.2)	38,119 (1.4)
Mental impairment	1,053 (0.1)	103,261 (11.3)	104,314 (4.0)
Renal function impairment	27,237 (1.6)	80,137 (8.7)	107,374 (4.1)
Heart function impairment	1,246 (0.1)	3,792 (0.4)	5,038 (0.2)
Respiratory function impairment	496 (0.1)	10,904 (1.2)	11,400 (0.4)
Liver function impairment	14,636 (0.9)	704 (0.1)	15,340 (0.6)
Facial deformity	1,492 (0.1)	1,244 (0.1)	2,736 (0.1)
Intestinal and urinary tract function impairment	16,264 (0.9)	806 (0.1)	17,070 (0.6)
Epilepsy	5,417 (0.3)	1,598 (0.2)	7,015 (0.3)

Values are weighted proportions representing the national population of adults with registered disabilities (*N* = 2,630,374)

Percentages in the Mild and Severe columns indicate the distribution of disability types within each severity group (column totals = 100%)

Total column percentages indicate the proportion of each disability type in the overall population

### Prevalence of unmet healthcare needs and relative factors

Among the study population, 17.3% reported unmet healthcare needs in the past year, representing approximately 456,000 individuals (weighted population estimate). The mean age of those with unmet healthcare needs was higher than those without unmet healthcare needs (66.63 vs. 62.63 years). Individuals aged ≥ 65 years had the highest prevalence (19.0%). Women reported higher unmet healthcare needs than male (20.3% vs. 15.2%) (Table 2).

**Table 2:** Characteristics of participants by unmet healthcare needs status (*N* = 2630,374)

Variables	Categories	Unmet healthcare needs (*n* = 456125, 17.3%)	No unmet healthcare needs (*n* = 2174248, 82.7%)
Predisposing factors			
Age		66.63 ± 17.32	62.63 ± 18.70
	19–34	16,285 (11.6%)	123,647 (88.4%)
	35–49	46,532 (17.2%)	223,413 (82.8%)
	50–64	114,196 (16.2%)	590,000 (83.8%)
	≥ 65	271,543 (19.0%)	1,156,148 (81.0%)
Sex	Male	230,876 (15.2%)	1,290,466 (84.8%)
	Female	225,249 (20.3%)	883,783 (79.7%)
Marital status	Married and cohabiting	200,569 (15.2%)	1,119,259 (84.8%)
	Married but not cohabiting or bereaved or divorced	173,243 (22.3%)	602,183 (77.7%)
	Unmarried	5,986 (30.7%)	13,495 (69.3%)
Education level	Elementary school or lower	197,851 (21.1%)	738,226 (78.9%)
	Middle school	79,012 (18.7%)	343,857 (81.3%)
	High school	115,728 (14.4%)	686,706 (85.6%)
	College or higher	63,534 (13.5%)	405,460 (86.5%)
Enabling factors			
Region	Seoul	53,036 (13.6%)	336,911 (86.4%)
	Metropolitan	126,839 (19.7%)	516,162 (80.3%)
	Others	276,249 (17.3%)	1,321,177 (82.7%)
Employment status	Employed	127,716 (13.6%)	810,571 (86.4%)
	Unemployed	323,154 (19.8%)	1,306,102 (80.2%)
Income	1Q (lowest)	112,843 (16.9%)	554,737 (83.1%)
	2Q	141,813 (21.9%)	506,789 (78.1%)
	3Q	128,407 (19.4%)	532,651 (80.6%)
	4Q (highest)	73,062 (11.2%)	580,071 (88.8%)
Medical insurance type	NHI	354,223 (16.2%)	1,835,399 (83.8%)
	Medicaid	100,670 (22.9%)	338,562 (77.1%)
	No/do not know	1,232 (81.1%)	288 (18.9%)
Going out alone	Possible	322,884 (14.9%)	1,838,741 (85.1%)
	Impossible	133,131 (28.4%)	335,508 (71.6%)
ADL dependency	1-4^b^	1.38 ± 0.62	1.17 ± 0.41
IADL dependency	1-4^b^	1.80 ± 0.90	1.46 ± 0.72
Vulnerable factors			
Disability severity	Mild severity	290,198 (16.9%)	1,422,440 (83.1%)
	Severe severity	165,927 (18.1%)	751,809 (81.9%)
Disability type	Physical disability	408,673 (17.9%)	1,872,632 (82.1%)
	Intellectual disability	47,452 (13.6%)	301,617 (86.4%)
Exercise	Yes	229,916 (13.3%)	1,498,146 (86.7%)
	No	226,209 (25.1%)	676,103 (74.9%)
Stress level	High	38,761 (29.0%)	95,037 (71.0%)
	Moderate	151,377 (22.6%)	518,294 (77.4%)
	Low	174,454 (14.7%)	1,013,192 (85.3%)
	None	87,581 (14.8%)	504,503 (85.2%)
Self-rated health status	Very good or good	50,343 (9.5%)	482,167 (90.5%)
	Moderate	110,272 (12.5%)	773,722 (87.5%)
	Bad or very bad	295,509 (24.3%)	918,360 (75.7%)
Depression	Yes	39,257 (26.7%)	107,752 (73.3%)
	No	6,225 (30.5%)	14,184 (69.5%)

Values are presented as unweighted n (weighted %). All percentages are weighted to reflect the national population of adults with registered disabilities (*N* = 2,630,374); unweighted sample sizes are provided for transparency

Statistical significance tests were not reported because nearly all comparisons reached statistical significance (*p*<0.001) due to the large sample size; clinical relevance should be judged by the magnitude of percentage differences. These bivariate associations do not account for confounding; see Table 4 for multivariable-adjusted results

*Q *quartile, *NHI *national health insurance, *ADL *activities of daily living, *IADL *instrumental activities of daily living

^a^Weighted proportions

^b^Scores range 1–4; higher scores indicate greater dependency

Unmet healthcare needs were more common among unmarried individuals (30.7%) and those with the lowest educational attainment (elementary school or lower: 21.1%). Regionally, the prevalence was highest among residents of metropolitan areas (19.7%), followed by other provinces (17.3%) and Seoul (13.6%). The unemployed group (19.8%) had a higher prevalence than the employed group (13.6%).

Across income quartiles, unmet healthcare needs were highest in the second quartile (21.9%), followed by the third (19.4%), first (16.9%), and fourth quartiles (11.2%). Individuals without or uncertain about insurance coverage reported markedly elevated levels of unmet healthcare needs (81.1%) compared with those covered by the NHI (16.2%) or Medicaid (22.9%).

Unmet healthcare needs were more frequent among people unable to go out alone (28.4%) and those with greater functional dependency (mean ADL score: 1.38 vs. 1.17; mean IADL score: 1.80 vs. 1.46). Individuals with severe disabilities had a slightly higher prevalence than those with mild disabilities (18.1% vs. 16.9%). Higher unmet healthcare needs were also observed among those who did not exercise (25.1%), those who reported high stress (29.0%), and those with poor or very poor self-rated health (24.3%).

### Reasons for self-reported unmet healthcare needs in people with disabilities

Reasons for unmet healthcare needs were classified according to Chen and Hou [14], which operationalized healthcare access framework proposed by Penchansky and Thomas [16]. Three dimensions were identified: accessibility (64.3%), availability (4.2%), and acceptability (31.5%).

With accessibility, transportation difficulties (36.5%) and financial cost (27.8%) were the most frequently cited barriers. Within availability, services not available in the area (2.6%) was the primary reason, followed by services not available when needed (0.8%) and waiting time too long (0.8%). Within acceptability, being too busy (13.0%), personal or family caregiving responsibilities (7.1%), perceived inadequacy of care (6.0%), and dislike or fear of healthcare providers (4.0%) were the main reasons (Table 3).

**Table 3:** Reasons for unmet healthcare needs among people with disability (*N* = 456,125)

Category	Reasons for unmet healthcare needs	*N* (%)
Accessibility		293,305 (64.3%)
	Transportation difficulties	166,407 (36.5%)
	Financial cost	126,898 (27.8%)
Availability		18,630 (4.2%)
	Services not available in area	11,654 (2.6%)
	Services not available when needed	3,539 (0.8%)
	Waiting time too long	3,437 (0.8%)
Acceptability		144,187 (31.5%)
	Too busy	59,458 (13.0%)
	Personal or family caregiving responsibilities	32,424 (7.1%)
	Dislike or fear of healthcare providers	18,754 (4.0%)
	Did not know where to seek care	3,917 (0.9%)
	Perceived inadequacy of care	27,383 (6.0%)
	Other	2,251 (0.5%)

Percentages are weighted to reflect the national population of adults with registered disabilities experiencing unmet healthcare needs (*N* = 456,125). Respondents selected one main reason; therefore, reasons and categories are mutually exclusive and sum to 100%. The classification into accessibility, availability, and acceptability was conducted post hoc for descriptive purposes and should be interpreted as pragmatic descriptive classifications rather than validated theoretical constructs

### Factors affecting unmet healthcare needs for people with disabilities

Multivariable logistic regression analysis was conducted to identify factors independently associated with unmet healthcare needs (Table 4).

**Table 4:** Factors associated with unmet healthcare needs by sex (*N* = 456,125)

Variables	Categories	Total (weighted *n* = 456,125)			Men (weighted *n* = 230,876/ 50.6%^a^)			Women (weighted *n* = 225,249/ 49.4%^a^)		
		OR	95% CI	*p*	OR	95% CI	*p*	OR	95% CI	*p*
Predisposing factors										
Age	19–34	0.99	0.97–1.02	0.734	1.18	1.15–1.22	< 0.001	0.64	0.61–0.67	< 0.001
	35–49	1.55	1.52–1.58	< 0.001	1.95	1.91–1.99	< 0.001	1.07	1.04–1.09	< 0.001
	50–64	1.21	1.20–1.23	< 0.001	1.45	1.43–1.47	< 0.001	0.97	0.96–0.99	< 0.001
	≥ 65	1.00			1.00			1.00		
Marital status	Married and cohabiting	0.91	0.90–0.92	< 0.001	1.05	1.04–1.07	< 0.001	0.71	0.69–0.73	< 0.001
	Married but not cohabiting or bereaved or divorced	1.14	1.12–1.15	< 0.001	1.40	1.38–1.43	< 0.001	0.79	0.77–0.81	< 0.001
	Unmarried	1.00			1.00			1.00		
Education level	Elementary school or lower	1.11	1.10–1.13	< 0.001	1.20	1.18–1.22	< 0.001	0.94	0.92–0.96	< 0.001
	Middle school	1.17	1.15–1.18	< 0.001	1.34	1.32–1.37	< 0.001	0.88	0.86–0.90	< 0.001
	High school	0.87	0.86–0.88	< 0.001	0.83	0.82–0.84	< 0.001	0.85	0.83–0.87	< 0.001
	College or higher	1.00			1.00			1.00		
Enabling factors										
Region	Seoul	1.00			1.00			1.00		
	Metropolitan	1.45	1.41–1.51	< 0.001	1.52	1.50–1.55	< 0.001	1.48	1.46–1.51	< 0.001
	Others	1.23	1.22–1.25	< 0.001	1.40	1.38–1.42	< 0.001	1.10	1.09–1.12	< 0.001
Employment status	Employed	1.00			1.00			1.00		
	Unemployed	1.15	1.13–1.17	< 0.001	1.18	1.15–1.20	< 0.001	1.20	1.16–1.24	< 0.001
Income	1Q (lowest)	1.42	1.40–1.44	< 0.001	1.91	1.87–1.94	< 0.001	1.12	1.10–1.15	< 0.001
	2Q	1.54	1.52–1.56	< 0.001	1.82	1.79–1.85	< 0.001	1.36	1.33–1.39	< 0.001
	3Q	1.41	1.39–1.43	< 0.001	1.26	1.24–1.28	< 0.001	1.62	1.58–1.65	< 0.001
	4Q (highest)	1.00			1.00			1.00		
Medical insurance type	NHI	1.00			1.00			1.00		
	Medicaid	1.18	1.16–1.19	< 0.001	1.12	1.11–1.14	< 0.001	1.21	1.19–1.23	< 0.001
	No/do not know	25.07	21.99–28.57	< 0.001	8.93	7.45–9.07	< 0.001	4.01	3.42–4.71	< 0.001
Going out alone	Possible	1.00			1.00			1.00		
	Impossible	1.11	1.09–1.12	< 0.001	0.92	0.90–0.94	< 0.001	1.27	1.24–1.29	< 0.001
ADL dependency	1-4^b^	0.96	0.95–0.97	< 0.001	1.13	1.11–1.14	< 0.001	0.84	0.83–0.86	< 0.001
IADL dependency	1-4^b^	1.50	1.49–1.51	< 0.001	1.63	1.61–1.65	< 0.001	1.38	1.35–1.38	< 0.001
Vulnerable factors										
Disability severity	Mild severity	1.00			1.00			1.00		
	Severe severity	0.86	0.85–0.86	< 0.001	0.73	0.72–0.74	< 0.001	1.04	1.03–1.05	< 0.001
Disability type	Physical disability	1.74	1.71–1.76	< 0.001	1.33	1.30–1.35	< 0.001	2.50	2.44–2.56	< 0.001
	Intellectual disability	1.00			1.00			1.00		
Smoking history	Non-smoker	1.00			1.00			1.00		
	Past-smoker	0.94	0.93–0.95	< 0.001	1.00	0.99–1.02	0.497	1.28	1.24–1.33	< 0.001
	Current-smoker	1.13	1.11–1.14	< 0.001	1.25	1.23–1.26	< 0.001	0.85	0.82–0.88	< 0.001
Alcohol consumption	Never drink	1.00			1.00			1.00		
	Less than 1 time per month	1.43	1.41–1.45	< 0.001	1.01	0.98–1.02	0.967	2.20	2.16–2.25	< 0.001
	1–4 times per month	0.88	0.87–0.89	< 0.001	0.97	0.96–0.99	< 0.001	0.65	0.64–0.67	< 0.001
	≥ 5 times per month	0.92	0.91–0.93	< 0.001	0.91	0.90–0.93	< 0.001	0.52	0.49–0.55	< 0.001
Exercise	Yes	1.00			1.00			1.00		
	No	1.53	1.51–1.54	< 0.001	1.55	1.53–1.56	< 0.001	1.53	1.51–1.54	< 0.001
Stress level	High	1.51	1.49–1.54	< 0.001	1.15	1.12–1.17	< 0.001	2.00	1.96–2.05	< 0.001
	Moderate	1.32	1.31–1.33	< 0.001	1.24	1.22–1.25	< 0.001	1.38	1.36–1.40	< 0.001
	Low	0.97	0.96–0.98	< 0.001	0.97	0.97–0.99	< 0.001	1.15	1.10–1.20	< 0.001
	None	1.00			1.00			1.00		
Self-rated health status	Very good or good	1.00			1.00			1.00		
	Moderate	1.12	1.11–1.13	< 0.001	1.14	1.12–1.15	< 0.001	1.18	1.15–1.21	< 0.001
	Bad or very bad	1.82	1.80–1.85	< 0.001	1.51	1.49–1.56	< 0.001	2.53	2.48–2.59	< 0.001
Depression	Yes	1.42	1.40–1.44	< 0.001	1.33	1.30–1.36	< 0.001	1.47	1.44–1.50	< 0.001
	No	1.00			1.00			1.00		

All values are weighted to reflect the national population of adults with registered disabilities experiencing unmet healthcare needs. Analyses accounted for the complex survey design using Taylor series linearization. Models were mutually adjusted for all variables shown in the table. Total models additionally adjusted for sex; sex-specific models did not adjust for sex. The “no insurance/do not know” group represents a relatively small subgroup; therefore, the large odds ratios should be interpreted with caution

*OR* odds ratio, *CI* confidence interval, *Q* quartile, *NHI* National Health Insurance, *ADL* activities of daily living, *IADL* instrumental activities of daily living

^a^Weighted proportions showing the percentage of each sex group among those with unmet healthcare needs

^b^Scores range 1–4; higher scores indicate greater dependency

#### Predisposing factors

Across the total sample, unmet healthcare needs were significantly higher in individuals aged 35–49 years (OR = 1.55; 95% CI, 1.52–1.58) and 50–64 years (OR = 1.21; 95% CI, 1.20–1.23) compared with those aged ≥ 65 years. Marital status was also associated with unmet healthcare needs: compared with unmarried individuals, those who were married but not cohabiting, bereaved, or divorced had higher unmet healthcare needs (OR = 1.14; 95% CI, 1.12–1.15), while those married and cohabiting had lower unmet needs (OR = 0.91; 95% CI, 0.90–0.92). Lower educational attainment was associated with higher unmet healthcare needs; participants with elementary education or below had higher unmet healthcare needs than those with college-level education (OR = 1.11; 95% CI, 1.10–1.13).

#### Enabling factors

All enabling factors were significant predictors. Residents of metropolitan areas had higher unmet healthcare needs than residents of Seoul (OR = 1.45; 95% CI, 1.41–1.51). Unemployment was associated with higher unmet healthcare needs (OR = 1.15; 95% CI, 1.13–1.17). Compared with the highest income quartile, unmet needs were elevated in the first (OR = 1.42; 95% CI, 1.40–1.44), second (OR = 1.54; 95% CI, 1.52–1.56), and third quartiles (OR = 1.41; 95% CI, 1.39–1.43). The “no insurance/do not know” group exhibited markedly elevated odds of unmet healthcare needs (OR = 25.07; 95% CI, 21.99–28.57). This category included 1,232 individuals (unweighted; 18% of the analytic sample), of whom 81.1% reported unmet healthcare needs compared with 16.2% among those with NHI. Given the high prevalence of unmet needs in this group and potential for quasi-complete separation in logistic regression, we conducted sensitivity analysis using Firth penalized logistic regression, which adjusts for sparse data bias. The Firth regression estimate was consistent with the main analysis (OR = 23.84; 95% CI, 20.91–27.19), supporting the stability of the estimates; detailed results are presented in Supplementary Table S5. The magnitude of association remained substantial in sex-specific analyses (men: OR = 8.93; women: OR = 4.01).

#### Vulnerable factors

All vulnerability-related variables were significant. Participants with severe disabilities had slightly lower unmet healthcare needs than those with mild disabilities (OR = 0.86; 95% CI, 0.85–0.86). This finding remained consistent across stratified analyses by income level, employment status, and insurance type; detailed subgroup results are presented in Supplementary Table S6. Those with physical disabilities had higher unmet healthcare needs than those with intellectual disabilities (OR = 1.74; 95% CI, 1.71–1.76). Individuals who did not engage in exercise reported higher unmet needs (OR = 1.53; 95% CI, 1.51–1.54). Both high stress (OR = 1.51; 95% CI, 1.49–1.54) were associated with higher unmet needs compared with low or no stress. Compared with participants reporting very good or good self-rated health, those reporting poor or very poor health had higher unmet needs (OR = 1.82; 95% CI, 1.80–1.85). Depression was associated with higher unmet healthcare needs (OR = 1.42; 95% CI, 1.40–1.44).

## Discussion

This study examined factors associated with unmet healthcare needs among people with disabilities in South Korea using nationally representative data (*N* = 6,772; weighted 2.63 million). Approximately 17.3% reported unmet healthcare needs—lower than prior estimates (32.4% in 2017 Korea [3], 40% in Europe [8]) but substantial given near-universal insurance coverage (> 97%). This persistent gap reveals a fundamental tension: universal insurance ensures financial protection without guaranteeing effective access, challenging policy assumptions that equate coverage expansion with access equity [6, 9, 20].

Applying Andersen’s Behavioral Model [18], unmet healthcare needs arose from predisposing, enabling, and vulnerability factors. Critically, accessibility barriers—transportation difficulties (36.5%) and financial cost (27.8%)—accounted for 64.3% of unmet healthcare needs (Table 3), underscoring that access failures often precede facility arrival [7, 21]. This predominance of pre-facility barriers suggests disability healthcare policy must shift from medical models toward socioecological frameworks recognizing how built environment, transportation, and economic structures shape access [5, 22].

Middle-aged adults (35–49) had 55% higher odds of unmet needs (OR = 1.55) than older adults (≥ 65), likely reflecting competing work and caregiving demands [3, 23]. Lower educational attainment predicted higher odds through reduced health literacy and less flexible employment [24, 25]. Absence of a cohabiting spouse increased odds by 9–14%, revealing structural dependencies wherein healthcare systems implicitly assume family support availability, systematically disadvantaging unmarried or socially isolated individuals [8, 22].

Metropolitan residents outside Seoul had higher unmet healthcare needs despite greater healthcare resource availability, challenging assumptions that urban settings necessarily provide better access to care. One possible explanation is the geographic concentration of disability-specialized infrastructure in Seoul, including barrier-free certified facilities and specialized rehabilitation services, compared with secondary metropolitan areas [17, 26]. In addition, high population density may create demand saturation, while complex transportation systems requiring multiple transfers may further impede access for individuals with mobility limitations [7, 21, 27]. These findings suggest that healthcare resource density alone may not ensure effective access for people with disabilities when services and infrastructure are insufficiently adapted to functional and mobility-related needs. Thus, crude supply indicators such as provider-to-population ratios may inadequately capture disability-specific accessibility [3].

Insurance status emerged as the most powerful predictor. Uninsured/uncertain individuals had 25-fold higher odds (OR = 25.07; 81.1% with unmet needs), confirmed by Firth regression (OR = 23.84; Supplementary Table S5). In near-universal coverage systems, remaining uninsured is unlikely to represent a simple absence of insurance alone. Rather, these individuals may include socially marginalized populations experiencing compounded vulnerabilities, including unstable administrative engagement, limited health system literacy, cognitive or communication impairments, unstable living situations, or weak linkage to welfare and healthcare institutions [2, 12]. Thus, insurance status in this context may function not only as a financing variable but also as a marker of broader social and institutional exclusion. Even Medicaid beneficiaries experienced higher unmet healthcare needs than National Health Insurance enrollees, suggesting that financial coverage alone may be insufficient to eliminate barriers related to provider accessibility, administrative complexity, and broader socioeconomic vulnerability [27, 28].

Greater IADL dependency predicted 50% higher odds (OR = 1.50), highlighting how access depends fundamentally on instrumental activities—managing finances, using transportation, organizing tasks. Healthcare systems designed assuming patient independence may systematically disadvantage those with IADL limitations, creating access failures rooted in design rather than individual deficits [3, 7]. In contrast, ADL dependency showed a weak inverse association (OR = 0.96) requiring cautious interpretation given its high correlation with IADL (VIF = 2.64–2.58; Supplementary Table S4). This likely reflects a suppression effect from simultaneously including two related measures; sensitivity analyses confirmed ADL shows the expected positive association when not controlling for IADL (Supplementary Table S3). Substantively, individuals with severe ADL limitations may be more consistently embedded in formal caregiving systems, whereas IADL limitations directly impede healthcare navigation without triggering equivalent support. This finding suggests that healthcare accessibility may be more strongly influenced by instrumental and navigational capacity than by basic self-care limitations alone.

Physical disability showed the second-largest effect (OR = 1.74), likely reflecting mobility barriers [7]. Effects varied substantially by sex, with comparatively smaller effects among men (OR = 1.32) and markedly stronger associations among women (OR = 2.48). High stress showed similar disparities (OR = 2.00 vs. 1.15 for men). These findings suggest an intersectional vulnerability in which gender-related caregiving roles, time constraints, and physical disability interact to create compounded structural barriers to healthcare access. Women with disabilities may face not only mobility-related limitations but also additional constraints related to caregiving responsibilities and reduced flexibility in seeking care [29, 30]. Such barriers may be further amplified by potential differences in provider responsiveness to women’s health concerns [31, 32]. These patterns persist even within universal coverage systems, indicating that insurance coverage alone may be insufficient to ensure equitable healthcare access for women with disabilities.

Severe disabilities were associated with 14% lower odds (OR = 0.86) of unmet healthcare needs compared to mild disabilities—counterintuitive yet consistent across income, employment, and insurance subgroups (OR range: 0.81–0.92; Supplementary Table S6), suggesting that the finding is unlikely to be fully explained by socioeconomic confounding. The pattern was most pronounced among low-income individuals (OR = 0.81) and Medicaid beneficiaries (OR = 0.83)—groups where support systems would be expected to have the greatest impact—consistent with the possibility that disability support programs are more effectively targeted toward individuals with greater functional impairments [3].

However, this suggests a concerning “policy blind spot” for mild disabilities. While experiencing meaningful limitations and barriers, individuals with mild disabilities may fall outside eligibility thresholds for support programs, encounter potential provider dismissiveness, and maintain higher expectations without adaptation—factors that may collectively contribute to disproportionately high perceived unmet needs [4, 5]. This finding is simultaneously encouraging (support systems function as intended for severe disabilities) and cautionary (systematic underservice of mild disabilities).

The large sample size (2.63 million weighted) provided substantial statistical power, although statistical significance does not uniformly translate into practical relevance. High-magnitude predictors (no insurance OR = 25.07, poor health OR = 1.82, physical disability OR = 1.74) represent clear priorities. Sensitivity analyses confirmed robustness: excluding proxy responses (12%) yielded all ORs within ± 5% of the main analysis; detailed results are presented in Supplementary Table S2. VIF values < 3.0 confirmed model stability (Supplementary Table S4). The 17.3% prevalence falls mid-range of international estimates (12–35%), suggesting common challenges translating coverage into access across universal systems.

### Policy implications

First, effort should prioritize strengthening transportation support (addressing 36.5% of unmet needs) through expanded paratransit capacity, simplified reservation systems, priority scheduling, extended service hours, and improved healthcare facility integration. Second, financial protection should be enhanced (27.8% of unmet needs) via copayment reductions, expanded covered benefits, and out-of-pocket spending caps. Third, target insurance enrollment assistance for highly vulnerable uninsured individuals (81.1% unmet needs) is warranted through embedded navigators, simplified processes, and consideration of automatic enrollment mechanisms where feasible. Fourth, addressing service gaps among individuals with mild disabilities may require broader eligibility criteria and intermediate support tiers. Fifth, sex-specific interventions may help address compounded barriers experienced by women. Sixth, infrastructure improvements should be prioritized in metropolitan areas outside Seoul.

### Limitations

First, this measure primarily reflects perceived access barriers based on individual experiences rather than comprehensive healthcare need. Self-reported unmet needs may not fully capture the severity or specific types of healthcare needs and do not differentiate the intensity, frequency, or clinical importance of unmet needs. Additionally, responses are subject to individual perception thresholds that may vary across disability severity. The categorization of reasons for unmet healthcare needs into accessibility, availability, and acceptability dimensions was conducted post hoc for descriptive purposes rather than being embedded in the original survey design. Accordingly, the acceptability category was broad and heterogeneous, and some conceptual overlap between categories may exist because several response options reflected subjective perceptions of healthcare access barriers.

Second, proxy responses (12%) may introduce measurement error or misclassification, although sensitivity analysis indicated minimal impact (Supplementary Table S2). Third, the small uninsured sample (*n* = 1,232) limited precision, although Firth regression confirmed robustness of estimate (Supplementary Table S5). Fourth, the cross-sectional design precludes causal inference and limits the ability to determine the direction of associations; therefore, reverse causality cannot be excluded. For example, unmet healthcare needs may contribute to poorer mental health outcomes, such as increased stress and depression, meaning psychosocial factors may act as both determinants and consequences of unmet healthcare needs. Finally, residual confounding from unmeasured factors (e.g., health literacy, discrimination, and social networks) cannot be excluded, and data collected in 2023 may not fully capture COVID-19 pandemic-related disruptions [33].

While our findings describe disparities in unmet healthcare needs, they do not allow definitive conclusions regarding equity.

## Conclusions

Among people with disabilities in South Korea, 17.3% experienced unmet healthcare needs despite near-universal coverage. Key factors included predisposing (age, marital status, education), enabling (income, insurance, geography, mobility, IADL), and vulnerability factors (disability type, behaviors, psychosocial distress). Women showed substantially stronger effects for physical disability (OR = 2.48 vs. 1.32), stress (OR = 2.00 vs. 1.15), and poor health (OR = 2.53 vs. 1.51). Severe disability paradoxically predicted lower unmet needs across subgroups (OR range: 0.81–0.92), demonstrating successful support targeting but revealing mild disability service gaps. Findings demonstrate coverage alone is insufficient—transportation support, financial protection, mild disability services, sex-specific interventions, and geographic redistribution are essential for genuine access equity.

## Supplementary Information

Supplementary Material 1.

## Data Availability

The data used in this study were obtained from the publicly available dataset provided by the Ministry of Health and Welfare of South Korea. Researchers can access the dataset upon submitting a research proposal and required documentation through the official data request platform.
