# Supplementary material for: Unmet healthcare needs among adults with disabilities in South Korea: a nationally representative analysis using the behavioral model
Source: BMC Public Health. 2026 Jun 26;26:2873. doi: 10.1186/s12889-026-28057-x (PMC13647642; doi:10.1186/s12889-026-28057-x)
Supplement: Supplementary file 1 [file 12889_2026_28057_MOESM1_ESM.docx]

**Supplementary Table S1. Comparison of Participants With Complete vs. Incomplete Data**

| **Characteristic** | **Complete Data (n=6,772)** | **Missing Data (n=1,228)** | **p-value** |
| --- | --- | --- | --- |
| Unmet healthcare needs, n (%) | 1,172 (17.3) | 218 (17.8) | 0.42 |
| Severe disability, n (%) | 2,465 (36.4) | 440 (35.8) | 0.38 |
| Male sex, n (%) | 3,426 (50.6) | 628 (51.1) | 0.56 |
| Age, mean (SE), years | 63.5 (0.3) | 64.1 (0.4) | 0.31 |

Note: Values are presented as unweighted n (%) for categorical variables and weighted mean (standard error, SE) for continuous variables. All percentages and means account for survey weights.

Comparisons were conducted using design-based chi-square tests (categorical variables) and design-based t-tests (continuous variables), accounting for the complex survey design. P-values test the null hypothesis of no difference between complete and missing data groups.

**Supplementary Table S2. Sensitivity Analysis Excluding Proxy-Reported Responses: Factors Associated with Unmet Healthcare Needs (N=400,140)**

| **Variables** | **Categories** | **Total**  **(weighted n=400,140)** | | | **Men**  **(weighted n=203,370 / 50.8%**ᵃ**)** | | | **Women**  **(weighted n=196,770 / 49.2%**ᵃ**)** | | |
| --- | --- | --- | --- | --- | --- | --- | --- | --- | --- | --- |
|  |  | **OR** | **95% CI** | ***p*** | **OR** | **95% CI** | ***p*** | **OR** | **95% CI** | ***p*** |
| **Predisposing factors** | | | | | | | | | | |
| Age | 19-34 | 0.99 | 0.97-1.02 | .734 | 1.18 | 1.15-1.22 | <.001 | 0.64 | 0.61-0.67 | <.001 |
|  | 35-49 | 1.54 | 1.51-1.57 | <.001 | 1.94 | 1.90-1.98 | <.001 | 1.07 | 1.04-1.10 | <.001 |
|  | 50-64 | 1.21 | 1.20-1.23 | <.001 | 1.45 | 1.43-1.47 | <.001 | 0.97 | 0.96-0.99 | <.001 |
|  | ≥ 65 | 1.00 |  |  | 1.00 |  |  | 1.00 |  |  |
| Marital status | Married and cohabiting | 0.91 | 0.90-0.92 | <.001 | 1.05 | 1.04-1.07 | <.001 | 0.71 | 0.69-0.73 | <.001 |
|  | Married but not cohabiting or bereaved or divorced | 1.14 | 1.12-1.15 | <.001 | 1.40 | 1.38-1.42 | <.001 | 0.79 | 0.77-0.81 | <.001 |
|  | Unmarried | 1.00 |  |  | 1.00 |  |  | 1.00 |  |  |
| Education level | Elementary school or lower | 1.11 | 1.09-1.13 | <.001 | 1.19 | 1.17-1.21 | <.001 | 0.94 | 0.92-0.96 | <.001 |
|  | Middle school | 1.16 | 1.14-1.18 | <.001 | 1.33 | 1.31-1.36 | <.001 | 0.88 | 0.86-0.90 | <.001 |
|  | High school | 0.87 | 0.86-0.88 | <.001 | 0.83 | 0.82-0.84 | <.001 | 0.85 | 0.83-0.87 | <.001 |
|  | College or higher | 1.00 |  |  | 1.00 |  |  | 1.00 |  |  |
| **Enabling factors** | | | | | | | | | | |
| Region | Seoul | 1.00 |  |  | 1.00 |  |  | 1.00 |  |  |
|  | Metropolitan | 1.46 | 1.42-1.50 | <.001 | 1.52 | 1.49-1.55 | <.001 | 1.48 | 1.46-1.51 | <.001 |
|  | Others | 1.23 | 1.22-1.25 | <.001 | 1.40 | 1.38-1.42 | <.001 | 1.10 | 1.09-1.12 | <.001 |
| Employment status | Employed | 1.00 |  |  | 1.00 |  |  | 1.00 |  |  |
|  | Unemployed | 1.15 | 1.13-1.17 | <.001 | 1.18 | 1.15-1.20 | <.001 | 1.20 | 1.16-1.24 | <.001 |
| Income | 1Q (lowest) | 1.42 | 1.40-1.44 | <.001 | 1.91 | 1.87-1.94 | <.001 | 1.12 | 1.10-1.15 | <.001 |
|  | 2Q | 1.53 | 1.51-1.55 | <.001 | 1.81 | 1.78-1.84 | <.001 | 1.36 | 1.33-1.39 | <.001 |
|  | 3Q | 1.41 | 1.39-1.43 | <.001 | 1.26 | 1.24-1.28 | <.001 | 1.61 | 1.57-1.65 | <.001 |
|  | 4Q (highest) | 1.00 |  |  | 1.00 |  |  | 1.00 |  |  |
| Medical insurance type | NHI | 1.00 |  |  | 1.00 |  |  | 1.00 |  |  |
|  | Medicaid | 1.18 | 1.16-1.19 | <.001 | 1.12 | 1.11-1.14 | <.001 | 1.21 | 1.19-1.23 | <.001 |
|  | No/do not know | 24.85 | 21.75-28.42 | <.001 | 8.88 | 7.42-10.62 | <.001 | 3.98 | 3.39-4.68 | <.001 |
| Going out alone | Possible | 1.00 |  |  | 1.00 |  |  | 1.00 |  |  |
|  | Impossible | 1.11 | 1.09-1.12 | <.001 | 0.92 | 0.90-0.94 | <.001 | 1.27 | 1.24-1.29 | <.001 |
| ADL dependency | 1-4b | 0.96 | 0.95-0.97 | <.001 | 1.13 | 1.11-1.14 | <.001 | 0.84 | 0.83-0.86 | <.001 |
| IADL dependency | 1-4^b^ | 1.50 | 1.49-1.51 | <.001 | 1.62 | 1.60-1.64 | <.001 | 1.38 | 1.36-1.40 | <.001 |
| **Vulnerable factors** | | | | | | | | | | |
| Disability severity | Mild severity | 1.00 |  |  | 1.00 |  |  | 1.00 |  |  |
|  | Severe severity | 0.87 | 0.86-0.88 | <.001 | 0.73 | 0.72-0.74 | <.001 | 1.04 | 1.03-1.05 | <.001 |
| Disability type | Physical disability | 1.72 | 1.69-1.75 | <.001 | 1.32 | 1.29-1.35 | <.001 | 2.48 | 2.42-2.54 | <.001 |
|  | Intellectual disability | 1.00 |  |  | 1.00 |  |  | 1.00 |  |  |
| Smoking history | Non-smoker | 1.00 |  |  | 1.00 |  |  | 1.00 |  |  |
|  | Past-smoker | 0.94 | 0.93-0.95 | <.001 | 1.00 | 0.99-1.02 | 0.497 | 1.28 | 1.24-1.33 | <.001 |
|  | Current-smoker | 1.13 | 1.11-1.14 | <.001 | 1.25 | 1.23-1.26 | <.001 | 0.85 | 0.82-0.88 | <.001 |
| Alcohol consumption | Never drink | 1.00 |  |  | 1.00 |  |  | 1.00 |  |  |
|  | Less than 1 time per month | 1.43 | 1.41-1.45 | <.001 | 1.01 | 0.99-1.03 | .967 | 2.19 | 2.15-2.24 | <.001 |
|  | 1-4 times per month | 0.88 | 0.87-0.89 | <.001 | 0.97 | 0.96-0.99 | <.001 | 0.65 | 0.64-0.67 | <.001 |
|  | ≥5 times per month | 0.92 | 0.91-0.93 | <.001 | 0.91 | 0.90-0.93 | <.001 | 0.52 | 0.49-0.55 | <.001 |
| Exercise | Yes | 1.00 |  |  | 1.00 |  |  | 1.00 |  |  |
|  | No | 1.52 | 1.50-1.54 | <.001 | 1.54 | 1.52-1.56 | <.001 | 1.52 | 1.50-1.54 | <.001 |
| Stress level | High | 1.50 | 1.48-1.53 | <.001 | 1.15 | 1.12-1.17 | <.001 | 1.99 | 1.95-2.04 | <.001 |
|  | Moderate | 1.32 | 1.31-1.33 | <.001 | 1.24 | 1.22-1.25 | <.001 | 1.38 | 1.36-1.40 | <.001 |
|  | Low | 0.97 | 0.96-0.98 | <.001 | 0.98 | 0.96-0.99 | <.001 | 0.97 | 0.95-0.99 | <.001 |
|  | None | 1.00 |  |  | 1.00 |  |  | 1.00 |  |  |
| Self-rated health status | Very good or good | 1.00 |  |  | 1.00 |  |  | 1.00 |  |  |
|  | Moderate | 1.12 | 1.11-1.13 | <.001 | 1.14 | 1.12-1.15 | <.001 | 1.18 | 1.15-1.21 | <.001 |
|  | Bad or very bad | 1.81 | 1.79-1.84 | <.001 | 1.50 | 1.48-1.53 | <.001 | 2.51 | 2.46-2.57 | <.001 |
| Depression | Yes | 1.41 | 1.39-1.43 | <.001 | 1.33 | 1.30-1.36 | <.001 | 1.47 | 1.44-1.50 | <.001 |
|  | No | 1.00 |  |  | 1.00 |  |  | 1.00 |  |  |

Note: Analysis restricted to self-reported responses only (n=5,956 unweighted; 88% of analytic sample), excluding 816 proxy-reported cases (12%). Weighted n=400,140 represents adults with registered disabilities who self-reported their healthcare experiences. All models adjusted for complex survey design using Taylor series linearization. Models were mutually adjusted for all variables shown. Total models additionally adjusted for sex; sex-specific models did not adjust for sex.

Results are substantively consistent with the main analysis (Table 4, n=6,772), with all effect estimates within ±5% and overlapping confidence intervals, confirming robustness to proxy response exclusion.

The largest difference was observed for severe disability (OR=0.87 vs. 0.86 in main analysis), representing a 1.2% change.

OR = odds ratio; CI = confidence interval; Q = quartile; NHI = National Health Insurance; ADL = activities of daily living; IADL = instrumental activities of daily living.

ᵃWeighted proportions showing the percentage of each sex group among those with unmet healthcare needs in the proxy-excluded sample.

**Supplementary Table S3. Sensitivity Analysis: Alternative Coding Methods for ADL and IADL Dependency**

| **Variable** | **Coding Method** | **OR (95% CI)**  **for Unmet Healthcare Needs** | **p-value** |
| --- | --- | --- | --- |
| ADL Dependency |  |  |  |
|  | Continuous (per-1-point increase) | 0.96 (0.95-0.97) | <.001 |
|  | Categorical (Quartiles) |  |  |
|  | Q1 (lowest dependency; reference) | 1.00 | — |
|  | Q2 | 1.04 (1.01-1.07) | .008 |
|  | Q3 | 1.08 (1.05-1.11) | <.001 |
|  | Q4 (highest dependency) | 1.12 (1.08-1.16) | <.001 |
|  | Dichotomized |  |  |
|  | Independent (reference) | 1.00 | — |
|  | Dependent (score ≥ 2) | 1.09 (1.06-1.12) | <.001 |
| IADL Dependency |  |  |  |
|  | Continuous (per-1-point increase) | 1.50 (1.49-1.51) | <.001 |
|  | Categorical (Quartiles) |  |  |
|  | Q1 (lowest dependency; reference) | 1.00 | — |
|  | Q2 | 1.38 (1.35-1.41) | <.001 |
|  | Q3 | 1.66 (1.63-1.69) | <.001 |
|  | Q4 (highest dependency) | 2.01 (1.97-2.05) | <.001 |
|  | Dichotomized |  |  |
|  | Independent (reference) | 1.00 | — |
|  | Dependent (score ≥ 2) | 1.48 (1.46-1.51) | <.001 |

Note: All models adjusted for age, sex, marital status, education, region, employment, income, insurance type, disability severity, disability type, going out alone, smoking, alcohol consumption, exercise, stress, self-rated health, and depression. Complex survey design weights and variance estimation applied. IADL results are consistent across methods. ADL continuous (OR=0.96) differs from categorical (OR=1.12) due to collinearity with IADL (VIF=2.64 and 2.58).

**Supplementary Table S4. Variance Inflation Factors for Predictor Variables**

| **Variable** | **VIF** | **Tolerance** |
| --- | --- | --- |
| Age (years) | 1.42 | 0.70 |
| Sex | 1.18 | 0.85 |
| Marital status | 1.67 | 0.60 |
| Education level | 2.21 | 0.45 |
| Region | 1.34 | 0.75 |
| Employment status | 2.78 | 0.36 |
| Income (quartiles) | 2.82 | 0.35 |
| Medical insurance type | 2.15 | 0.46 |
| Going out alone | 2.08 | 0.48 |
| ADL dependency | 2.64 | 0.38 |
| IADL dependency | 2.58 | 0.39 |
| Disability severity | 2.31 | 0.43 |
| Disability type | 2.26 | 0.44 |
| Smoking history | 1.52 | 0.66 |
| Alcohol consumption | 1.41 | 0.71 |
| Physical exercise | 1.38 | 0.72 |
| Stress level | 1.87 | 0.53 |
| Self-rated health | 2.11 | 0.47 |
| Depression | 1.94 | 0.52 |

Note: VIF = variance inflation factor; Tolerance = 1/VIF. VIF values >5 indicate potential multicollinearity concerns; VIF >10 indicates severe multicollinearity. All VIF values were below 3.0 (range: 1.18–2.82), indicating no evidence of problematic collinearity. The highest VIF values were observed for income (2.82) and employment status (2.78), which is expected given their natural correlation, but remain well within acceptable limits.

**Supplementary Table S5. Sensitivity Analysis for Insurance Status: Comparison of Standard and Firth Penalized Logistic Regression**

| **Analysis Method** | **OR for No Insurance/Don’t Know** | **95% CI** |
| --- | --- | --- |
| Standard logistic regression | 25.07 | 21.99-28.57 |
| Firth penalized regression | 23.84 | 20.91-27.19 |
| Excluding no insurance category | Not applicable (reference category: NHI) | - |
| Medicaid only | 1.18 | 1.16-1.19 |

Note: Firth penalized regression addresses potential bias when outcome prevalence is extreme (no insurance group: 81.1% unmet needs; n=1,232). Results confirm robustness (Firth OR=23.84 vs. standard OR=25.07).

**Supplementary Table S6. Stratified Analysis of the Association Between Disability Severity and Unmet Healthcare Needs**

| **Stratifying Variable** | **Severe vs. Mild Disability OR (95% CI)** | **p-value** |
| --- | --- | --- |
| Income level |  |  |
| Q1 (lowest) | 0.81 (0.79-0.83) | <.001 |
| Q2 | 0.84 (0.82-0.86) | <.001 |
| Q3 | 0.87 (0.85-0.89) | <.001 |
| Q4 (highest) | 0.92 (0.89-0.95) | <.001 |
| Employment status |  |  |
| Employed | 0.88 (0.85-0.91) | <.001 |
| Unemployed | 0.84 (0.83-0.86) | <.001 |
| Insurance type |  |  |
| NHI | 0.87 (0.86-0.88) | <.001 |
| Medicaid | 0.83 (0.81-0.85) | <.001 |

Note: All models adjusted for age, sex, marital status, education, region, ADL/IADL dependency, disability type, health behaviors, and health status. Each row shows results from a separate stratified analysis. The protective effect of severe disability (OR <1.0) was consistent across all subgroups, with the strongest effects observed in lower-income groups (OR=0.81) and Medicaid beneficiaries (OR=0.83), suggesting that targeted support systems may effectively reduce access barriers for those with greater functional limitations.
